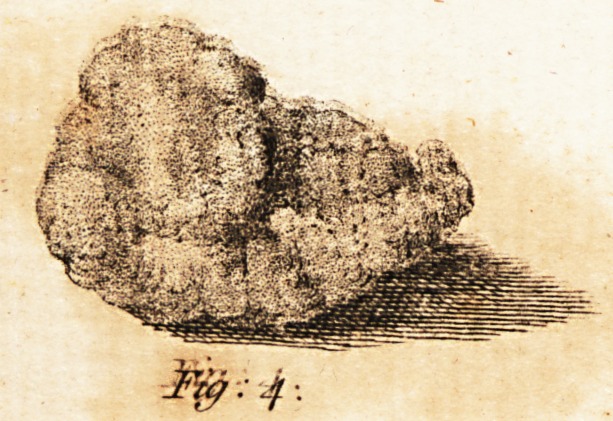# Account of a Calculus Extracted from a Cyst in the Neck

**Published:** 1790

**Authors:** Edward Ford

**Affiliations:** Surgeon of the Westminster General Dispensary


VII. *Account of a Calculus extracted from a Cyst in the Neck. By the same.*

THE patient from whose neck this stone * was taken, was a healthy girl of twelve years of age. The account which I received from her mother was, that for two years before there had been a small moveable swelling, appearing like an enlarged gland; that it had been attended with no pain, but that lately it had grown very hard. It was situated on the left side of the neck over the platysma myoides muscle, and was removed by dissecting it with the cyst, from which it was impossible to separate it. The wound healed up in a few days by the application of a little sticking plaster.

The concretion seems to be of a chalky nature, composed of several small stones held together by the membrane surrounding them: it resembles evidently the calculi sometimes found in the lungs.

* See figure 4 of the annexed plate.

## Figures and Tables

**Fig: 4. f1:**